# Bidirectional Synaptic Plasticity Is Driven by Sex Neurosteroids Targeting Estrogen and Androgen Receptors in Hippocampal CA1 Pyramidal Neurons

**DOI:** 10.3389/fncel.2019.00534

**Published:** 2019-12-04

**Authors:** Alessandro Tozzi, Valentina Durante, Paolo Manca, Michela Di Mauro, Juan Blasi, Silvarosa Grassi, Paolo Calabresi, Suguru Kawato, Vito Enrico Pettorossi

**Affiliations:** ^1^Department of Experimental Medicine, Section of Physiology and Biochemistry, University of Perugia, Perugia, Italy; ^2^Department of Medicine, Section of Neurological Clinic, “Santa Maria della Misericordia” Hospital, University of Perugia, Perugia, Italy; ^3^Department de Patologia i Terapèutica Experimental, Facultat de Medicina, Campus de Bellvitge, Universitat de Barcelona, Barcelona, Spain; ^4^Department of Cognitive Neuroscience, Faculty of Pharma-Science, Teikyo University, Tokyo, Japan; ^5^Department of Biophysics and Life Sciences, Graduate School of Arts and Sciences, The University of Tokyo, Tokyo, Japan

**Keywords:** estrogen, androgen, synaptic plasticity, long-term potentiation, long-term depression

## Abstract

Neuroactive estrogenic and androgenic steroids influence synaptic transmission, finely modulating synaptic plasticity in several brain regions including the hippocampus. While estrogens facilitate long-term potentiation (LTP), androgens are involved in the induction of long-term depression (LTD) and depotentiation (DP) of synaptic transmission. To examine sex neurosteroid-dependent LTP and LTD in single cells, patch-clamp recordings from hippocampal CA1 pyramidal neurons of male rats and selective antagonists for estrogen receptors (ERs) and androgen (AR) receptors were used. LTP induced by high-frequency stimulation (HFS) depended on activation of ERs since it was prevented by the ER antagonist ICI 182,780 in most of the neurons. Application of the selective antagonists for ERα (MPP) or ERβ (PHTPP) caused a reduction of the LTP amplitude, while these antagonists in combination, prevented LTP completely. LTP was never affected by blocking AR with the specific antagonist flutamide. Conversely, LTD and DP, elicited by low-frequency stimulation (LFS), were impeded by flutamide, but not by ICI 182,780, in most neurons. In few cells, LTD was even reverted to LTP by flutamide. Moreover, the combined application of both ER and AR antagonists completely prevented both LTP and LTD/DP in the same neuron. The current study demonstrates that the activation of ERs is necessary for inducing LTP in hippocampal pyramidal neurons, whereas the activation of ARs is required for LTD and DP. Moreover, both estrogen- and androgen-dependent LTP and LTD can be expressed in the same pyramidal neurons, suggesting that the activation of sex neurosteroids signaling pathways is responsible for bidirectional synaptic plasticity.

## Introduction

The sex steroids 17β-estradiol (E2), testosterone (T) and 5α-dihydrotestosterone (DHT) participate in the rapid modulation of long-term potentiation (LTP) and long-term depression (LTD) in different brain areas (McEwen, 2002; Isgor and Sengelaub, 2003; MacLusky et al., 2006; Hajszan et al., 2008; Grassi et al., 2011, 2013; Pettorossi et al., 2013; Scarduzio et al., 2013; Di Mauro et al., 2015, 2017; Tozzi et al., 2015). This modulation may involve membrane receptors for E2 (ERs) and androgens (ARs; Kerr et al., 1995; Kalita et al., 2005; Milner et al., 2005; Revankar et al., 2005; Tabori et al., 2005; Pedram et al., 2006; Foradori et al., 2008; Morissette et al., 2008; Raz et al., 2008; Levin, 2009). The influence of sex steroids on synaptic plasticity is exerted by either the circulating steroids of gonadal origin or steroids synthesized in the nervous system (neurosteroids; Baulieu, 1997; Compagnone and Mellon, 2000) through conversion of T into E2 and DHT by P450-aromatase and 5α-reductase enzymes, respectively (Selmanoff et al., 1977; Simpson et al., 1994; Kimoto et al., 2001; Mukai et al., 2006; Hojo et al., 2008, 2009).

Within the central nervous system (CNS) sex steroids are present at reasonable concentrations (Selmanoff et al., 1977; Kimoto et al., 2001; Hojo et al., 2004, 2008, 2009) since they can be synthesized *de novo* by neuronal activity (Balthazart et al., 2001; Kimoto et al., 2001; Hojo et al., 2004, 2008, 2009; Balthazart and Ball, 2006; Mukai et al., 2006; Ooishi et al., 2012a). In the hippocampus, for example, E2, T and DHT levels are much higher than in the plasma (Hojo et al., 2009; Kato et al., 2013) allowing synaptic modulation both in males and females (Selmanoff et al., 1977; Kimoto et al., 2001; Hojo et al., 2004, 2008, 2009; Mukai et al., 2006). Accordingly, sex neurosteroids play a relevant role on hippocampal bidirectional synaptic plasticity, with E2 facilitating LTP and DHT promoting LTD or depotentiation (DP) of synaptic transmission by stimulating ERs or ARs respectively (Grassi et al., 2011; Pettorossi et al., 2013; Di Mauro et al., 2015, 2017). These neurosteroids can also influence the function of the hippocampal network exerting neurotrophic effects by promoting dendritic spine formation (Vierk et al., 2014; Hasegawa et al., 2015; Hatanaka et al., 2015; Fester et al., 2016; Hojo and Kawato, 2018). In order to test whether neurosteroid-mediated bidirectional synaptic changes are dependent on distinct hippocampal pyramidal neurons or on a more heterogeneous pool of cells, as suggested by our previous recordings of field potentials, we performed whole-cell patch-clamp recordings from single CA1 hippocampal pyramidal neurons of male rats. We also tested the influence of ER and AR activation on LTP and LTD/DP, induced by high and low-frequency stimulation (LFS) protocols, respectively.

## Materials and Methods

### Ethic Statement on Animal Use

All procedures were conducted in conformity with the European Directive 2010/63/EU, in accordance with protocols approved by the Animal Care and Use Committee at the University of Perugia (Italy) and by the Italian Ministry of Health (D.lgs 26/2014, authorization n. 297/2016-PR). Wistar rats (Charles River Laboratories, Wilmington, MA, USA) were kept (two per cage) under regular lighting conditions (12 h light/dark cycle) and given food and water *ad libitum*. All efforts were made to minimize the number of animals used and their suffering.

### Electrophysiology

Wistar male rats, at P30 for the analysis of the effect of sex neurosteroid receptors blockade and at P80–90 to validate the study in sexually mature rats, were sacrificed under deep anesthesia by cervical dislocation. To avoid any possible influence of cyclic, systemic estrogenic fluctuation on the induction of synaptic plasticity only male rats were used (Warren et al., 1995; Good et al., 1999). The brain was rapidly removed and immersed for 2–3 min in ice-cold artificial cerebrospinal fluid (aCSF; in mM: 126 NaCl, 2.5 KCl, 1.2 MgCl_2_, 1.2 NaH_2_PO_4_, 2.4 CaCl_2_, 10 glucose, 25 NaHCO_3_) continuously bubbled with 95% O_2_ and 5% CO_2_, pH 7.4. After hippocampus extraction, 250 μm-thick transverse slices were cut in ice-cold aCSF with a vibratome (Vibratome, series 1000 plus, St. Louis, MO, USA) and allowed to recover in aCSF, bubbled with an O_2_ 95% and CO_2_ 5% gas mixture at room temperature for 1–2 h before experimental recordings.

**Figure 1:**
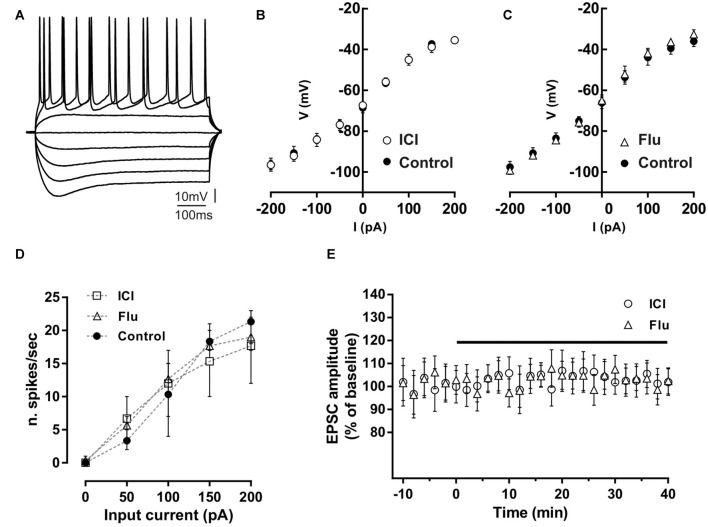
Effect of ICI 182,780 and flutamide on basal membrane properties of hippocampal CA1 neurons. **(A)** Voltage traces of a hippocampal CA1 neuron acquired following depolarizing and hyperpolarizing steps of currents of 50 pA.** (B,C)** Current-voltage plots of neurons recorded in control conditions, in the presence of 100 nM ICI 182,780 (ICI) or 100 nM flutamide (Flu). **(D)** Input-output graph showing the average number of action potentials per second evoked by depolarizing steps of currents in neurons in control conditions or in the presence of ICI or flutamide. **(E)** Time-course graph showing the excitatory postsynaptic current (EPSC) amplitude of neurons recorded before and during bath application of ICI or flutamide for 40 min.

A single slice was transferred to a recording chamber and submerged in a continuously flowing aCSF (34°C; 2.5–3 ml/min) bubbled with a 95% O_2_–5% CO_2_ gas mixture. Neurons were visualized using differential interference contrast (Nomarski) and infrared microscopy (Olympus). All the examined neurons were pyramidal cells (PCs) located in the CA1 hippocampal region. PCs were visually and electrophysiologically identified by their resting membrane potential (~65 mV), low frequency of action potentials following injection of positive steps of current and the presence of a sag potential (h-current) at hyperpolarizing steps of currents (Figure 1). Whole-cell voltage-clamp recordings (Vhold −70 mV) were performed with borosilicate glass pipettes (4–7 MΩ; Ra 15–30 MΩ) filled with a standard internal solution containing (in mM): 125 K^+^ -gluconate, 0.1 CaCl_2_, 2 MgCl_2_, 0.1 EGTA, 10 HEPES, adjusted to pH 7.3 with KOH. Signals were amplified with a Multiclamp 700B amplifier (Molecular Devices), recorded and stored on personal computer using pClamp 10 (Molecular Devices). Pipette resistances ranged from 3.5 to 5 MΩ. Membrane currents were continuously monitored and access resistance was in the range of 15–30 MΩ before electric compensation (60%–80% routinely used). Excitatory postsynaptic currents (EPSCs) were evoked by a bipolar electrode, connected to a stimulation unit (Grass Telefactor), positioned on the hippocampal slice in the stratum radiatum zone to stimulate Schaffer collaterals fibers (testing stimuli 0.1 Hz, intensity 10–20 V, 30–40 μs), The recording electrode was placed in the stratum pyramidale of the CA1 area. Evoked EPSCs were recorded for 10 min to obtain a stable baseline and then for an additional 30–40 min after the stimulation for long-term changes of synaptic transmission. LTP was induced by high-frequency stimulation (HFS) consisting of three trains of stimuli of 3 s (20 s inter-train interval) at 100 Hz. For LTD or DP of synaptic transmission LFS at 1 Hz for 15 min was applied. DP was induced by an LFS protocol applied 15–25 min after HFS.

### Drugs

Drugs were applied by dissolving them to the desired final concentrations in the aCSF and by switching the perfusion from control solution to drug-containing solution. Drugs applied in the recording chamber were delivered for at least 10 min before the induction of long-term synaptic effects and maintained throughout the experiment. The ER antagonist ICI 182,780 (ICI, 100 nM), the selective antagonists for ERα MPP (1 μM) and ERβ PHTPP (1 μM), and the AR antagonist flutamide (Flu, 100 nM) were purchased from Tocris-Cookson (Bristol, UK).

### Histochemistry

Rat hippocampus was fixed immediately after extraction by immersion in 4% paraformaldehyde (PFA) in phosphate-buffered saline (PBS; 100 mM, pH 7.2) for 12 h. Samples were cryoprotected by immersion in 30% sucrose, embedded in OCT media and snap-frozen in precooled isobutanol. Cryostat sections (10 μm thickness) were obtained, mounted onto poly-lysine coated microscope slides (Superfrost Plus, Thermo Fisher Scientific) and stored at 20°C until used. Some slices were stained with hematoxylin and eosin for light microscopy analysis. The rest of the slices were processed for triple immunofluorescence staining. The sections were incubated with 20% fetal bovine serum, 0.2% gelatin and 0.2% Triton X-100 in PBS for 1 h at RT to reduce non-specific binding followed by an overnight incubation with the primary antibodies at 4°C, in PBS containing 0.2% gelatin, 0.2% Triton X-100 and 1% fetal bovine serum. The primary antibodies used were from Abcam (Cambridge, UK): rat monoclonal antibody to AR (ref. ab2742, 1:100 dilution), mouse monoclonal antibody to ER-alpha (ref. ab2746, 1:50, dilution) and rabbit polyclonal to ER-beta (ref. ab3577, 1:1,000, dilution). After three washes with PBS, sections were incubated for 1 h at RT with Alexa Fluor 488 goat anti-rat antibodies, Alexa Fluor 555 donkey anti-rabbit antibodies and Alexa 647 goat anti-mouse antibodies (Molecular Probes, at 1:500 dilution). After three washes with PBS, sections were mounted with Immunofluore mounting medium (ICN) and observed with a Leica TCS-SL Spectral confocal microscope (Centres Científics I Tecnològics, Universitat de Barcelona).

### Statistical Analysis

Electrophysiology data analysis was performed off-line using Clampfit 10 (Molecular Devices) and GraphPad Prism 5 (GraphPad Software, San Diego, CA, USA). Values given in the text and figures are mean ± SE, *n* representing the number of recorded neurons. Only one neuron per slice was recorded. Changes of EPSC amplitude induced by drugs or by stimulation protocols were expressed as a percentage of the baseline, which represents the normalized EPSC mean amplitude acquired during a stable period (10–15 min) before delivering drugs or stimulation. In each experiment, the occurrence of LTP or LTD was statistically verified by the student’s *t*-test for unpaired samples by comparing the value of EPSC amplitude measured at the end of the recording (30–40 min) after the stimulation to the baseline amplitude. To prove the induction of DP we compared in each experiment the pre-LFS EPSC values with those measured 20 min after LFS using the student’s *t-test* for unpaired samples. Possible changes induced by drugs on membrane excitability and synaptic responses were statistically examined by the student’s *t*-test for paired samples comparing values 5 min before and 10–15 min after drug application. The significance of the difference observed in the occurrence of the LTP, LTD, DP and the absence of effect was established by using observed vs. expected *X*^2^ tests. The comparison of the amplitude of the long-term effects between different experimental groups was performed using the unpaired student’s *t*-test or one-way ANOVA followed by the Tukey *post hoc* test was used to compare groups presented in the dot plot graphs. Two-way ANOVA was also performed to verify the influence of drugs on the basal membrane electrical properties of CA1 pyramidal neurons (control/drug and voltage at different step current). The significance level was established at *p* < 0.05.

## Results

### Effect of ER or AR Antagonism on the Basal Membrane Properties and Basal Synaptic Responses of CA1 Pyramidal Neurons

Possible effects of ER and AR blockade on the basal membrane properties of hippocampal CA1 PC were firstly analyzed by whole-cell patch-clamp recording. The firing pattern discharge and current-voltage relationship were analyzed applying hyperpolarizing and depolarizing steps of currents to neurons in control condition and in the presence of the ER antagonist ICI or the AR antagonist flutamide (Figures 1A–D). Current-voltage relationship was not altered by these drugs (control vs. ICI, *n* = 5; two-way ANOVA, *F*_(1,71)_ = 0.01, *p* = 0.92; control vs. flutamide, *n* = 5; two-way ANOVA, *F*_(1,81)_ = 0.38, *p* = 0.54). The firing pattern discharge was neither affected in the presence of ICI or Flu (one-way ANOVA, *F*_(2,12)_ = 0.010, *p* = 0.99, Figure 1D). These data suggest that both ER and AR activation does not affect the basal membrane electrical properties of CA1 pyramidal neurons. Moreover, the application of either ICI (*n* = 5) and flutamide (*n* = 5) to the slices did not also alter *per se* the EPSC amplitude (student’s *t*-test, *p* = 0.22–0.35; Figure 1E). The finding that either the ER or AR blockade has no effect on the membrane electrical properties and on the synaptic response of CA1 PCs seems to exclude an influence of estrogenic and androgenic signals in regulating the basal neuronal excitability and synaptic responsiveness.

### Effects of ER and AR Blockade on LTP Induction

**Figure 2:**
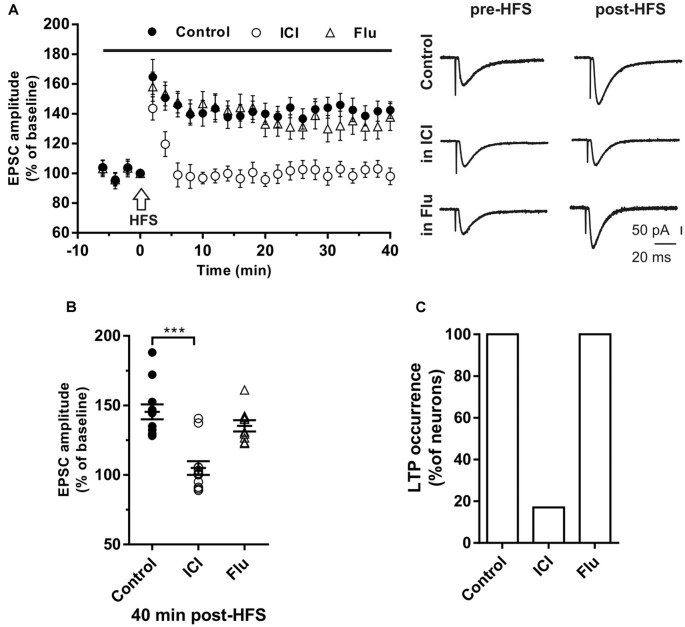
Effects of ICI 182,780 and flutamide on long-term potentiation (LTP). **(A)** Time-course (left) of the EPSC amplitude before and after the application of the HIGH-frequency stimulation (HFS) protocol (arrow) in control and under ER or AR blocking agents (horizontal black bar). Representative traces (right) of EPSCs recorded in control conditions (top), in the presence of ICI (middle), or Flu (bottom), before (left) and 40 min after the application of the HFS protocol. **(B)** Dot plot showing the EPSC amplitudes of all neurons measured 40 min post-HFS in control-condition, in the presence of ICI and Flu. **(C)** Histogram showing the percentage of neurons (occurrence) showing LTP. Control, *n* = 12; ICI, *n* = 12, Flu, *n* = 10. Note that in the presence of ICI LTP was abolished in most of the neurons. ***p < 0.001.

LTP was first induced in control conditions by applying the HFS protocol to stimulate the Schaffer collaterals fibers in the slices. In young rats (P30) LTP occurred in all recorded neurons (*n* = 12) since the EPSC amplitude measured 40 min after the HFS was significantly increased in all neurons. The amplitude of LTP relative to baseline was 142.4 ± 5.6% (Figures 2A,B). To test the role of E2 on the LTP induction of hippocampal CA1 neurons, we applied the HFS protocol to a group of neurons in the presence of the ER antagonist ICI. LTP was prevented in 10 out of 12 neurons. In fact, in these neurons, the EPSC amplitude was not significantly changed following the HFS (individual neurons: pre- vs. post-stimulus, student’s *t*-test, *p* = 0.8–0.4). In the remaining neurons, LTP was observed with an amplitude of 145 and 140% (pre- vs. post-stimulus student’s *t*-test, *p* < 0.05–0.01). The occurrence of LTP was significantly reduced by ICI application (ICI vs. control, *X*^2^ = 60, *df* = 2, *p* < 0.0001; Figure 2C).

To test the role of androgens on LTP induction a group of neurons were recorded in the presence of the AR antagonist flutamide. Similar to what observed in control conditions, when HFS was delivered under flutamide, LTP of similar amplitude was induced (137.8 ± 9.2%) in all tested neurons (*n* = 10; occurrence: flutamide vs. control, *X*^2^ = 0.40, *df* = 2, *p* = 0.81; LTP amplitude: flutamide vs. control, student’s *t*-test, *t* = 1.28, *p* = 0.72; Figure 2C).

We examined the influence of ER blockade also in a group of older rats (P80–90). We found that the LTP was induced in all neurons (*n* = 6; amplitude 146.6 ± 8.3%; occurrence 100%) but not in the presence of ICI (*n* = 4). Conversely, the application of flutamide (*n* = 4) did not affect the LTP (amplitude 141.7 ± 10.6%; occurrence 100%, data not shown).

### Effect of ERα and ERβ Blockade on LTP Induction

**Figure 3:**
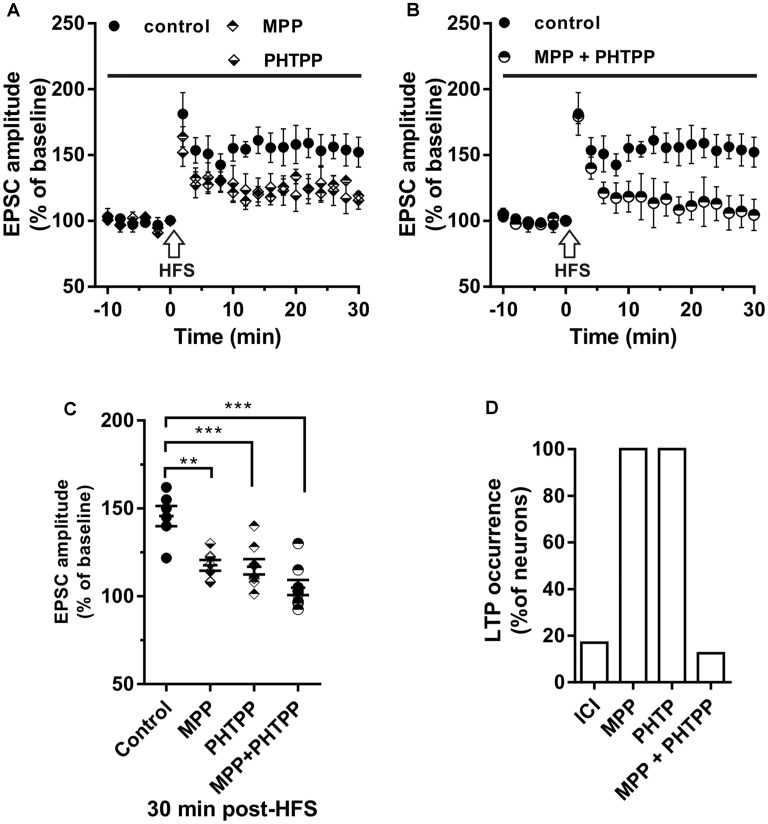
Effect of ERα antagonist MPP and ERβ antagonist PHTPP on LTP. **(A,B)** Time-course of the EPSC amplitude before and after the application of HFS in control and under separate **(A)** and combined **(B)** ERα and ERβ antagonists (drug application period: horizontal line). **(C)** Dot plot showing the EPSC amplitudes of all neurons measured 30 min post-HFS in control-condition, in the presence of MPP, PHTPP, and MPP plus PHTPP. **(D)** Percentage of LTP occurrence under ICI, MPP and under combined MPP and PHTPP. Control, *n* = 12, ICI, *n* = 12, MPP *n* = 7, PHTPP, *n* = 8, MPP plus PHTPP, *n* = 8. Note that similar partially reduced LTP was observed under MPP and PHTPP, while LTP was abolished under the application of both the drugs, as occurred under ICI. **p < 0.01. ***p < 0.001.

To evaluate the contribution of ERα and ERβ on the LTP induction, the HFS protocol was delivered in the presence of the ERα antagonist MPP (*n* = 7) or ERβ antagonist PHTPP (*n* = 8). In the presence of MPP, LTP was induced in all neurons but its amplitude (125.33 ± 15.11%) was significantly smaller compared to control (MPP vs. control, student’s *t*-test, *t* = 2.27, *P* < 0.05; Figure 3A). Similarly, also under PHTPP a small LTP of 121.3 ± 11.32% was induced by HFS in all neurons (PHTPP vs. control, student’s *t*-test, *t* = 2.29, *p* < 0.05). No significant difference was observed in the amplitude of LTP measured under MPP or PHTPP (MPP vs. PHTPP, student’s *t*-test, *t* = 0.51, *p* = 0.62; Figure 3A). Moreover, in the presence of both the ERα and ERβ antagonist, no LTP was induced by the HFS in seven out of eight neurons, while one cell presented LTP (Figures 3C,D). Statistical analysis revealed that the reduction of the LTP occurrence in the presence of ICI and of MPP plus PHTPP was not significantly different (reduction of 2 out of 10 neurons for ICI; one out of eight neurons for MPP plus PHTPP; occurrence comparison, *X*^2^ = 2, 28, *df* = 2, *p* = 0.32; Figures 3B–D).

### Effects of ER and AR Blockade on LTD Induction

**Figure 4:**
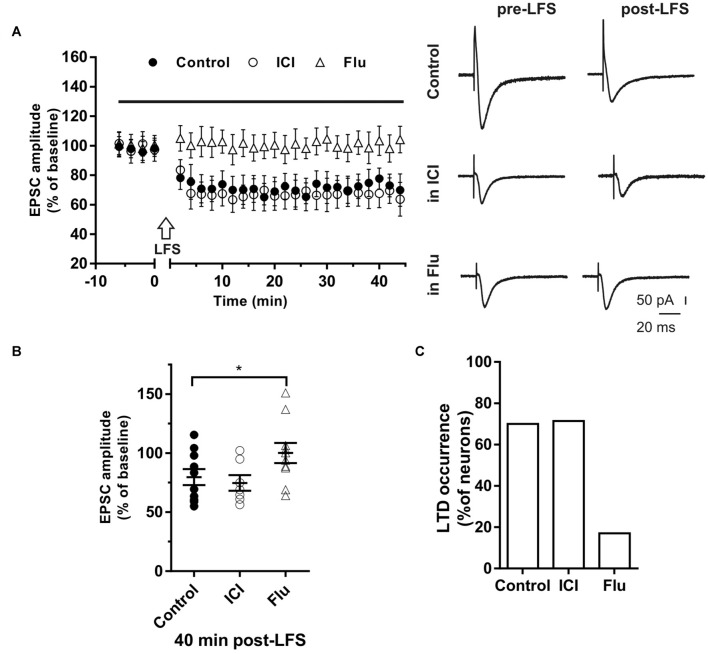
Effects of ICI 182,780 and flutamide on long-term depression (LTD). **(A)** Time-course (left) of the EPSC amplitude before and after the application of the low-frequency stimulation (LFS) protocol (arrow) in control and under ER or AR blocking agents (horizontal black bar). Representative traces (right) of EPSCs recorded in control conditions (top), in the presence of ICI (middle), or Flu (bottom), before (left) and 40 min after the application of the LFS protocol. **(B)** Dot plot showing the EPSC amplitudes of all neurons measured 40 min post-LFS in control-condition, in the presence of ICI and Flu. **(C)** Percentage of LTP occurrence. Control, *n* = 10, ICI, *n* = 7, Flutamide, *n* = 10. Note that the occurrence of LTD was remarkably reduced under the AR block. *p < 0.05.

The role of E2 and DHT was also tested on LTD induced in single hippocampal PCs by applying the LFS protocol. Neurons of young rats (P30) recorded in control conditions showed that the EPSC amplitude was significantly reduced to 67.41 ± 9.31% of the baseline in 7 out of 10 neurons, while no effect was observed in the remaining (Figures 4A,B). LFS application under ICI induced LTD of 64.4 ± 10.71% in five out of seven neurons with occurrence not significantly different from the control condition (ICI vs. control student’s *t*-test, *t* = 0.39, *p* = 0.70, *X*^2^ = 1.30, *df* = 2, *p* = 0.52; Figure 4).

In the presence of the AR antagonist flutamide, LTD was prevented in 10 out of 12 neurons (Figures 4A,B). In fact, in two neurons LFS induced LTD of 64% and 69% with a markedly decreased occurrence compared to control (*X*^2^ = 17.6, *df* = 2, *p* < 0.0005, Figure 4C). In particular, no change of the EPSC amplitude was observed in eight neurons while LTP was found in two cells (150.98% and 137.1%). Similarly, in older rats (P80–90), we found that LTD was induced in half of the neurons (four out of eight neurons; amplitude 66.6 ± 5.1%, *n* = 4) with lower occurrence compared to younger animals (*X*^2^ = 15.6, *df* = 2, *p* < 0.001). In the presence of flutamide, however, the occurrence of LTD was further reduced since it was prevented in six out of seven neurons (14%, *X*^2^ = 19.2, *df* = 2, *p* < 0.001). Conversely, the ER blocker ICI did not affect either the amplitude (ICI vs. control, student’s *t*-test, *t* = 1.09, *p* = 0.79) or the occurrence of LTD (6 out of 7 neurons, *X*^2^ = 1.11, *df* = 2, *p* = 0.42, not shown).

### Effect of ER and AR Blockade on DP of Synaptic Transmission

**Figure 5:**
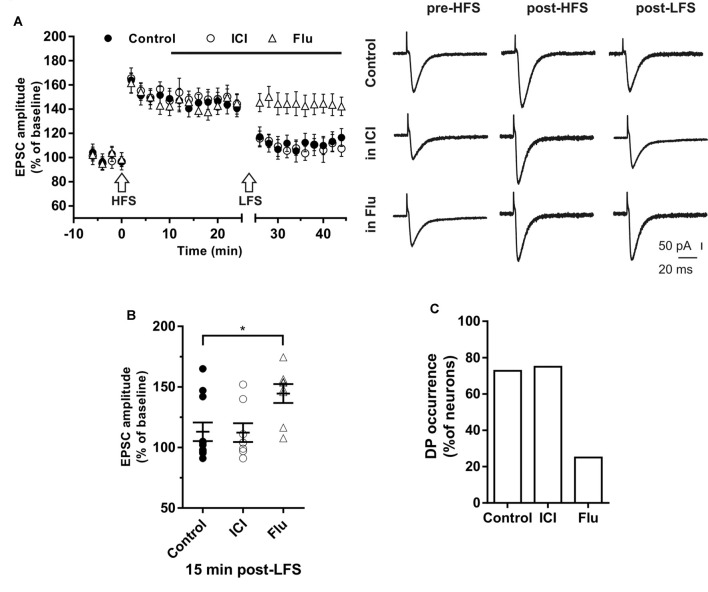
Effects of ICI 182,780 and flutamide on the depotentiation (DP) of LTP. **(A)** Time-courses (left) of the EPSC amplitudes before and after the application of the HFS and after the LFS protocols in control conditions, in the presence of ICI or Flu (horizontal black bar). Representative traces (right) of EPSCs recorded in control conditions (top), in the presence of ICI (middle), or Flu (bottom), before HFS (left), 15 min after HFS (center) and 15 min after the LFS protocol (right). **(B)** Dot plot showing the EPSC amplitudes of all neurons measured 15 min post-LFS in control-condition, in the presence of ICI and Flu. **(C)** Percentage of DP occurrence. Control, *n* = 11, ICI, *n* = 7, Flu, *n* = 8. Note that the occurrence of DP was remarkably reduced under AR blockade. *p < 0.05.

To analyze the effect of the possible involvement of ER and AR activity on DP, LTP was induced by HFS and, followed 30 min after, by LFS to induce DP. This paradigm was then repeated in the presence of ICI or flutamide to block ER or AR, respectively (Figures 5A,B).

In control conditions this procedure induced DP in 8 out of 11 neurons (Figure 5B), where the EPSC amplitude was reduced to the pre-HFS values (99.25 ± 3.16%). In the remaining three neurons, after LTP induction, the EPSC amplitude was not further modified by the subsequent LFS protocol (152.6 ± 8.76%, Figure 5B).

To study the effect of ER blockade on DP, LTP was firstly induced by the HFS protocol in a group of neurons (EPSC amplitude post-HFS, 149.72 ± 6.94%). Ten minutes later, ICI was continuously applied for the rest of the experiment. When LFS was applied to the slices 15 min after ICI application, DP was induced in six out of eight neurons. The EPSC amplitude was reduced to pre-HFS values (101.35 ± 4.21%). LFS did not induce DP in two neurons (144.6 ± 7.9%, Figures 5A,B). Accordingly, the occurrence of DP under ICI was not significantly changed respect to control conditions (ICI vs. control, *X*^2^ = 1.16, *df* = 2, *p* = 0.56; Figure 5C).

To explore the role of AR on the induction of DP, LTP was firstly induced in a group of neurons (158.91 ± 7.83%), then after 10 min flutamide was applied, and after subsequent 15 min, the LFS was delivered. In six out of eight neurons DP did not occur. In the remaining neurons, LFS induced DP (Figures 5A,B). These data show that the occurrence of DP was suppressed under AR blockade (flutamide vs. control, *X*^2^ = 19.5, *df* = 2, *p* < 0.0001; Figure 5C).

### Effect of Combined Blockade of ER and AR on LTP and Depotentiation

**Figure 6:**
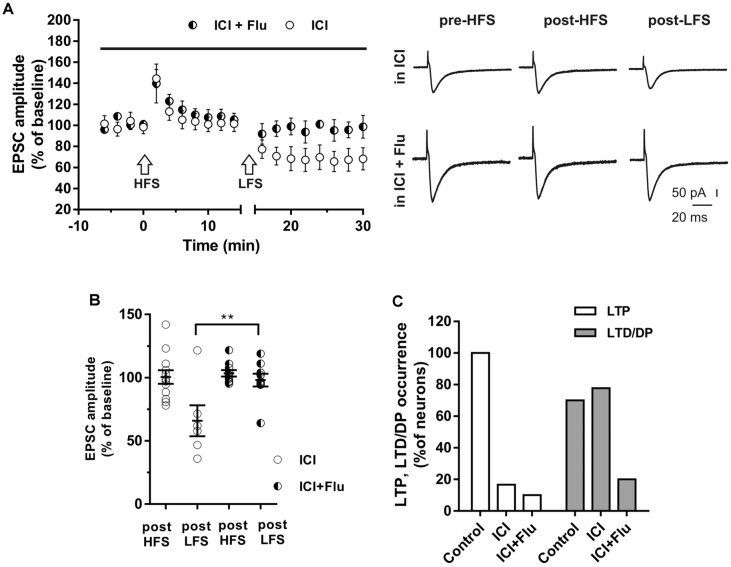
Effects of ICI 182,780 and flutamide on LTP and DP. **(A)** Time-courses (left) of the EPSC amplitudes before and after the application of the HFS and after the LFS protocols in the presence of ICI or in the presence of ICI plus Flu (horizontal black bar). Representative traces (right) of EPSCs recorded in ICI (top) or in ICI plus Flu (bottom), before HFS (left), 15 min after HFS (center) and 15 min after the LFS protocol (right). **(B)** Dot plot showing the EPSC amplitudes of all neurons measured 15 min post-HFS and 15 min post-LFS in the presence of ICI and ICI plus Flu. **(C)** Percentage of LTP occurrence. Control, *n* = 12, ICI, *n* = 10, ICI + Flu, *n* = 10). Note that in the neurons in which the LTP was present (control) the subsequent LFS induced DP of LTP, while in the neurons in which LTP was abolished by the ER blockade, LFS induced LTD. This LTD was almost abolished in the presence of the AR blockade. **p < 0.01.

To investigate whether E2 and DHT were able to influence long-term synaptic plasticity in the same neurons, ICI plus flutamide were applied to simultaneously block ER and AR and the HFS was delivered to induce LTP, followed by LFS to induce DP (Figure 6). The application of ICI and flutamide prevented the LTP induction in 9 out of 10 neurons of young rats (P30) and also prevented the subsequent DP in 8 of these latter neurons (Figure 6). Conversely, when ICI was applied alone, in 10 out of 12 neurons HFS induced no LTP (97.8 ± 9.66%) but the subsequent application of LFS was able to induce LTD in 7 out of these 10 neurons (61.34 ± 13.15%) and produced no effect in the remaining three neurons. Thus, the occurrence of LTD was significantly reduced in the presence of both ICI and flutamide compared with that under ICI (ICI plus flutamide vs. ICI, *X*^2^ = 15.6, *df* = 2, *p* < 0.0005) and it was similar to the occurrence of DP measured under flutamide alone (LTD under ICI plus flutamide vs. DP under flutamide, *X*^2^ = 0.5 *df* = 2, *p* = 0.78) or to that of LTD in control neurons under flutamide (LTD under ICI plus flutamide vs. LTD under flutamide, *X*^2^ = 0.62, *df* = 2, *p* = 0.73).

**Figure 7:**
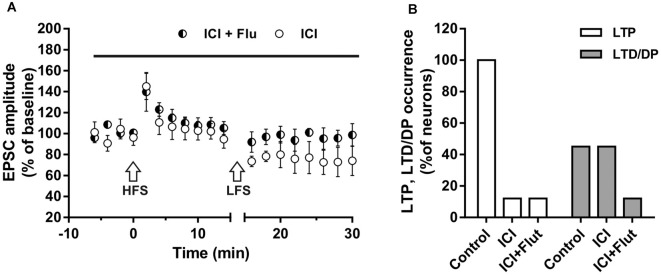
Effects of ICI 182,780 and flutamide on LTP and DP of adult rats. **(A)** Time-courses (left) of the EPSC amplitudes before and after the application of the HFS and after the LFS protocols in the presence of ICI or in the presence of ICI plus Flu (horizontal black bar). **(B)** Percentage of LTP occurrence. Control, *n* = 8, ICI, *n* = 8, ICI + Flu, *n* = 7.

We also examined the effects of ER and AR block on DP of a previously induced LTP in adult rats (P90). As for LTD, DP occurrence was lower than that measured in younger animals (four out of nine; *X*^2^ = 16.9, *df* = 2, *p* < 0.0005, not shown). In adult rats, we also examined the effects of ER and AR block on LTP and subsequent DP in the same neuron. We found that in the continuous presence of ICI, the HFS protocol did not induce LTP in any of the neurons (*n* = 8), whereas the subsequent LFS protocol induced LTD in four of them (paired *t*-test, *t*_(3)_ = 4.877, *p* < 0.05; Figure 7A). When ER and AR were blocked simultaneously by co-administering ICI and flutamide (*n* = 7), LTP was prevented while the depression was prevented in 6 of the neurons. The occurrence of LTD significantly decreased from 50 to 14.5% (*X*^2^ = 12.8, *df* = 2, *p* < 0.001, Figure 7B), not different from the occurrence of DP observed under flutamide alone (*X*^2^ = 0.75, *df* = 2, *p* = 0.74).

### Co-expression of ER and AR in Hippocampal CA1 Pyramidal Neurons

**Figure 8:**
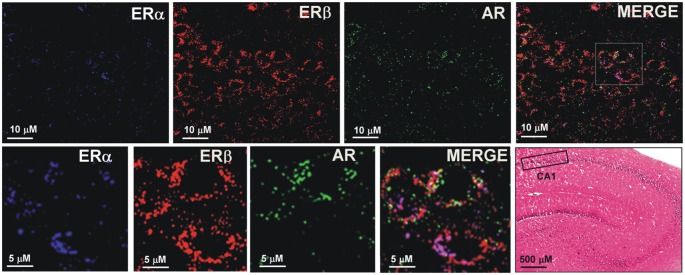
Immunofluorescence staining of ERα, ERβ and AR. Confocal images of triple immunostaining show the co-localization of ERα, ERβ, and AR in the same hippocampus CA1 neurons. The lower row shows a higher magnification of the area depicted in the MERGE image of the upper row. The Hematoxilin and Eosin staining on the left side of the lower row shows an image of the hippocampus at low magnification and the region analyzed by immunofluorescence (box in the CA1 region).

The sections from hippocampal slices were processed for triple immunofluorescence experiments to analyze the presence and possible colocalization of steroids receptors, in particular, ERα, ERβ, and AR, in CA1 pyramidal neurons. Confocal microscopy images show the cytoplasmic/nuclear immunostaining of pyramidal neurons for the three receptors (Figure 8). These results suggest the colocalization of ERα, ERβ and AR in the same pyramidal neurons, supporting the effect of neurosteroids in synaptic plasticity.

## Discussion

This study demonstrated that hippocampal neurons can express either LTP and LTD/DP of synaptic transmission, depending on the activation of ERs by E2 or ARs by DHT. Patch-clamp recordings of CA1 hippocampal pyramidal neurons revealed that ERs antagonism by ICI prevents LTP in the majority of the cells with no effect on LTD or DP. Moreover, both ERα and ERβ activation contributed to LTP induction since the ERα blocker MPP and the ERβ blocker PHTPP individually reduced the LTP, while LTP was fully prevented when these receptor antagonists were applied together. This complete block seems to exclude a direct involvement of other possible ER subtypes (Hadjimarkou and Vasudevan, 2018). In line with these results, the involvement of both ERα and ERβ in the E2-induced LTP has been previously shown using agonists of ERα and ERβ (Ooishi et al., 2012b).

The AR blocker flutamide prevented LTD/DP in most of the neurons with no effect on LTP induction and independently on the sexual maturation of the animal. These data are consistent with our previous observations based on field potentials recordings (Grassi et al., 2011, 2013; Pettorossi et al., 2013; Di Mauro et al., 2015, 2017) in which responses resulting from the activation of multiple and different types of neurons, such as PCs and interneurons, did not allow univocal neuronal identification. Conversely, recording from single neurons allowed neuronal identification revealing that sex neurosteroids are able to control LTP and LTD induction in hippocampal PCs of the CA1 region.

The analysis of synaptic responses obtained from recordings of single neurons showed that in the majority of the cells ER and AR activations are critical to inducing LTP and LTD, respectively. These findings differed from what observed in our previous reports based on field potential recordings, where ER antagonism induced only a reduction of the LTP amplitude, and the AR antagonist reverted LTD into an LTP of small amplitude. These differences might be explained by the fact that the small LTP previously reported in field potentials recordings in the presence of ICI or flutamide (Pettorossi et al., 2013) may result from different responsiveness of PCs to estrogenic or androgenic neurosteroids, since not in all the tested neurons ER and AR block affected the induction of long-term synaptic plasticity. Another reason for the observed difference could be the activation of different neuronal types, such as interneurons, as measured by field potential recordings, providing different responsiveness to neurosteroids.

**Figure 9:**
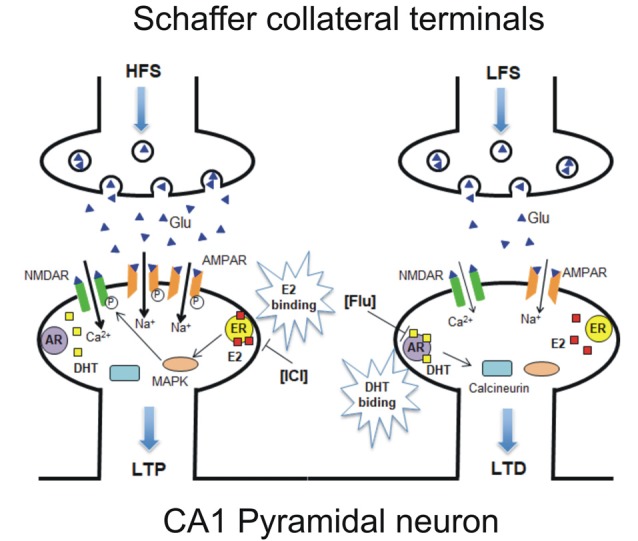
Model of a possible molecular mechanism in which estrogen and androgen influence the induction of LTP and LTD within the same neuron. (Left) HFS-induced LTP is established *via* E2 binding to synaptic ER upon HFS, leading to activation of MAPK and NMDAR pathways, resulting in an increase or phosphorylation of AMPARs. ICI-induced LTP suppression can be explained by the prevention of E2 binding to ER (Figure 2). (Right) LFS-induced LTD/DP is established *via* dihydrotestosterone (DHT) binding to synaptic AR upon LFS, leading to calcineurin activation and NMDAR-suppression pathways, resulting in a decrease or dephosphorylation of AMPARs. Flu-induced LTD suppression can be explained by the prevention of DHT binding to AR (Figure 4). It should be noted that E2 and DHT are endogenously synthesized in the hippocampus, although steroid synthesis enzymes are not drawn in the figure. Glu, Glutamate; NMDAR, N-methyl-D-aspartate type glutamate receptor; AMPAR, a-amino-3-hydroxy-5-methyl-4-isoxazole propionate type glutamate receptor.

The evidence of a variable responsiveness of hippocampal pyramidal neurons to estrogen and androgen opens the question of whether both ERs and ARs are expressed in the same hippocampal PC. Thus, single PCs were recorded in the presence of both ER and AR antagonists and HFS protocol, followed by LFS, was applied to induce LTP followed by LTD/DP in the same neuron. In these conditions, both opposite forms of synaptic plasticity were affected with ICI preventing LTP, and flutamide impeding LTD induction in most of the neurons, showing the same occurrence as that observed in naïve neurons under AR antagonist. This evidence strongly suggests that the cellular machinery at the basis of ER-dependent LTP and AR-dependent LTD are both present in most of the hippocampal PCs. As previously reported, the possible molecular mechanism responsible for the induction of ER-mediated synaptic plasticity for LTP induction requires the activation of MAPK and NMDAR pathways, while the AR-mediated LTD is based on the activation of calcineurin and suppression of NMDAR signaling (Figure 9; Hasegawa et al., 2015).

Our findings also show that the effect of ER and AR stimulation on bidirectional synaptic plasticity is not age-dependent, since both in juvenile (P30) and in sexually mature rats (P90) the block of ER and AR is able to prevent, in the same neuron, the induction of LTP and LTD, respectively. These data suggest that sex neurosteroids are relevant for shaping hippocampal synaptic plasticity throughout the life span, even though in adult rats the influence of sex neurosteroids appears to be milder, as the probability to induce LTD is lower in adult rats compared to the young animals even in absence of the blocking agent.

Moreover, sex-related differences in the influence of the sex neurosteroids on the induction of LTP have been previously reported (Grassi et al., 2012; Vierk et al., 2014) thus the occurrence of sex neurosteroid-dependent LTP and LTD is relevant and should be further explored also in neurons of female animals, examining possible changes related to the oestrous cycle.

The rapid suppression of LTP and LTD by blocking ERs and ARs implies the involvement of membrane-linked ERs- and ARs-activated intracellular pathways in PCs, since rapid modulation (within 30 min) of LTP and LTD is unlikely achieved by genomic modulation of nuclear ER and AR. Accordingly, hippocampal CA1 glutamatergic neurons are found to express ERs not only in nuclei/cytoplasm but also in pre- and post-synaptic elements (Milner et al., 2005; Mukai et al., 2006). In fact, it has been shown that some ERs are associated with the PSD, implying synaptic membrane binding of ERs (Boulware and Mermelstein, 2009; Meitzen et al., 2013). Therefore, membrane binding of ERs might occur in adult hippocampal neurons (Milner et al., 2005; Mukai et al., 2007, 2010; Hojo et al., 2008). ARs were also found to be expressed in CA1 neurons at the postsynaptic level and some of them specifically associated with PSD (Tabori et al., 2005; Hatanaka et al., 2015), suggesting that ARs are available to participate in androgen-induced LTD.

However, definitive evidence on ERs and ARs binding the membrane and on the balanced expression of ERs and ARs in the same neurons has not been obtained yet. For example, ERs and ARs have been described to be associated with the membrane by palmitoylation, as observed in peripheral cells such as epithelial cells or MCF7 cells (Pedram et al., 2007; Levin and Hammes, 2016). Our study provided evidence for ER and AR co-expression in the same pyramidal neurons, as shown by immunostaining. However, antibodies not only labeled membrane-bound ERs and ARs but also the cytosolic/nuclear receptors. Since this immunolabeling investigation is not conclusive, further, more specific analysis of membrane ER and AR is required.

E2 and DHT influence on bidirectional synaptic plasticity depends on the local activity of P450-aromatase and 5α-reductase. The expression of these synthesizing enzymes for E2 and DHT in the hippocampal CA1 neurons, strongly support the key role of local synthesis of estrogen and androgen in influencing synaptic plasticity (Vierk et al., 2014; Fester et al., 2016; Hojo and Kawato, 2018). This supports the interpretation of our electrophysiological results, suggesting that the synthesis of E2 by P450-aromatase and DHT by 5α-reductase take place in the same pyramidal neurons and their activity may vary depending on the neuron activity (Balthazart et al., 2001; Kimoto et al., 2001; Balthazart and Ball, 2006; Mukai et al., 2006; Hojo et al., 2008, 2009; Ooishi et al., 2012a; Pettorossi et al., 2013; Di Mauro et al., 2017).

In conclusion, this work provides evidence that single hippocampal pyramidal neurons express both ERs and ARs, whose activation is responsible for LTP or LTD/DP induction, respectively. Thus, depending on higher vs. lower frequency of afferent fibers activity, E_2_ or DHT may change their availability participating to LTP and LTD/DP of synaptic transmission.

## Data Availability Statement

The datasets generated for this study are available on request to the corresponding author.

## Ethics Statement

The animal study was reviewed and approved by Animal Care and Use Committee of the University of Perugia (Italy) and the Italian Ministry of Health (D.lgs 26/2014, authorization n. 297/2016-PR).

## Author Contributions

AT, SG and VP contributed to the conception and design of the study. AT, VD and MD performed the electrophysiological recordings. AT, VD, SG and VP performed the statistical analysis. PM and JB performed immunoistochemistry and wrote sections of the manuscript. AT and VP wrote the first draft of the manuscript. AT, VP, SK and PC provided substantial contributions to the interpretation of data for the work revising it critically for important intellectual content. All authors contributed to manuscript revision, read and approved the submitted version.

## Conflict of Interest

The authors declare that the research was conducted in the absence of any commercial or financial relationships that could be construed as a potential conflict of interest.
